# Anxiety disorders predict fasting to control weight: A longitudinal large cohort study of adolescents

**DOI:** 10.1002/erv.2714

**Published:** 2019-12-17

**Authors:** E. Caitlin Lloyd, Anne M. Haase, Stephanie Zerwas, Nadia Micali

**Affiliations:** ^1^ Centre for Exercise, Nutrition and Health Sciences University of Bristol Bristol UK; ^2^ Public Health Sciences Division Fred Hutchinson Cancer Research Centre Seattle Washington; ^3^ Department of Nutrition University of North Carolina at Chapel Hill Chapel Hill North Carolina; ^4^ Department of Psychiatry, Faculty of Medicine University of Geneva Geneva Switzerland; ^5^ Division of Child and Adolescent Psychiatry, Department of Child and Adolescent Health Geneva University Hospital Geneva Switzerland; ^6^ Great Ormond Street Institute of Child Health University College London London UK

**Keywords:** adolescent, ALSPAC, anorexia nervosa, anxiety disorders, eating behaviour

## Abstract

**Objective:**

To determine whether anxiety disorders are prospectively associated with fasting for weight‐loss/to avoid weight‐gain, a behaviour that precedes and is typical of anorexia nervosa (AN), during adolescence.

**Method:**

Participants were 2,406 female adolescents of the Avon Longitudinal Study of Parents and Children. Anxiety disorders were assessed when participants were aged 13–14 and 15–16; fasting was measured approximately 2 years after each anxiety assessment. Generalised estimating equation models examined whether anxiety disorders predicted later fasting, across the two longitudinal waves of data. To probe the moderating effect of time, data were stratified by wave and binary logistic regression analyses completed.

**Results:**

Across longitudinal waves, anxiety disorder presence predicted increased risk of later fasting. Evidence from wave‐stratified analyses supported a positive association between anxiety disorder presence at wave 15–16 and fasting at wave 17–18, however did not indicate an association between anxiety disorders at wave 13–14 and fasting at wave 15–16.

**Discussion:**

Anxiety disorder presence in mid‐late, but not early, adolescence predicted increased likelihood of later fasting. The differential association could be explained by anxiety being parent‐reported at wave 13–14. Findings highlight anxiety disorder pathology as a possible eating disorder prevention target, though the nature of association observed requires clarification.

## 1 INTRODUCTION

Anorexia nervosa (AN) is a severe eating disorder that has a range of adverse consequences for long‐term physical health (Mehler & Brown, 2015), and the highest mortality rate of any psychiatric disorder (Arcelus, Mitchell, Wales, & Nielsen, 2011). The defining feature of AN is persistent starvation (American Psychiatric Association [APA], 2013; Walsh, 2011), which is accompanied by significant fear of weight‐gain despite the maintenance of a very low weight. Lifetime prevalence of AN is estimated to be 3.64% amongst women (Micali, Martini, et al., 2017), with AN incidence highest during adolescence (Micali, Hagberg, Petersen, & Treasure, 2013). Generally time to recovery is protracted (Strober, Freeman, & Morrell, 1997; Zerwas et al., 2013), and a significant proportion of individuals experience severe and enduring AN (Broomfield, Stedal, Touyz, & Rhodes, 2017), meeting full diagnostic criteria for many years (Steinhausen, 2002).

**Highlights**

- Anxiety disorders predicted subsequent engagement in fasting for weight‐loss/to avoid weight‐gain, behaviour that precedes and is typical of anorexia nervosa, in a female adolescent sample.
- There was greater support for an association between anxiety disorders and fasting later in the course of adolescence.
- Findings highlight anxiety disorder pathology as potential intervention target in eating disorder prevention.

Various aetiological models propose that anxiety is a causal risk factor for the development of AN (Haynos & Fruzzetti, 2011; Kaye et al., 2003; Kaye, Fudge, & Paulus, 2009; Lloyd, Frampton, Verplanken, & Haase, 2017; Nunn, Frampton, & Lask, 2012). It is suggested that dietary restriction alleviates anxiety, meaning the behaviour has particularly favourable outcomes for individuals with high levels of anxiety. Such favourable outcomes encourage continued, and progressively more extreme, engagement in dietary restriction. Over time this leads to a dependence on dietary restriction, in particular for the management of anxiety that is increasingly focused on eating and weight‐gain, reflecting the presence of AN pathology.

Studies have addressed model hypotheses by probing associations between anxiety disorders and AN. Cross‐sectional associations are well characterised, with AN populations displaying increased rates of anxiety disorders as compared to populations of individuals without AN (Kaye et al., 2004; Swinbourne & Touyz, 2007). Cross‐sectional data cannot address questions of temporality however, and while retrospective studies report anxiety disorders to frequently precede AN onset (Swinbourne & Touyz, 2007), findings may be affected by recall bias (Jacobi, Hayward, de Zwaan, Kraemer, & Agras, 2004). One study using population registry data reported enhanced risk of subsequent AN in individuals diagnosed with anxiety disorders (Meier et al., 2015). However, in this study anxiety disorder and AN diagnoses were detected only if individuals received specialist care (i.e., beyond that of a general practitioner), which may have biased conclusions. In a nationally representative cohort, obsessive–compulsive disorder (OCD) predicted subsequent AN but there was no predictive effect of anxiety disorders (a category that does not include OCD or posttraumatic stress disorder [PTSD] in the latest diagnostic manuals) on later AN (Buckner, Silgado, & Lewinsohn, 2010). Notably the low prevalence of AN resulted in imprecise estimates in this study.

One approach to identifying factors predictive of relatively rare illnesses within community samples is to consider disorder symptoms, rather than diagnoses, as the outcome variables. The greater prevalence of symptoms, as compared with diagnoses, within a population means that under this approach studies are better able to accurately identify factors associated with the pathology of interest. Restrictive eating is a diagnostic criterion and core feature of AN (APA, 2013), but also precedes the onset of the disorder such that it is characterised as a prodromal symptom (Jacobi et al., 2004; Patton, Johnson‐Sabine, Wood, Mann, & Wakeling, 1990; Stice, Gau, Rohde, & Shaw, 2017). It may, then, be particularly advantageous to study predictors of restrictive eating behaviours typical of AN, in terms of identifying factors prospectively associated with disorder development. Furthermore, probing associations between anxiety and eating behaviour allows for a more direct assessment of the mechanistic hypothesis that anxiety increases risk of AN by encouraging continued and progressively more extreme engagement in dietary restriction.

A previous study reported no longitudinal predictive influence of anxiety symptoms on disordered eating (Zerwas, Von Holle, Watson, Gottfredson, & Bulik, 2014), however the specific association with dietary restriction was not assessed. Fasting for weight‐loss or to avoid weight‐gain is an extreme form of food avoidance that exists across the eating disorders, but is most prevalent in AN (Aardoom, Dingemans, Slof Op't Landt, & Van Furth, 2012; Linardon et al., 2018), and associated with greater AN severity (De Young et al., 2013). A recent investigation observed a prospective association between certain latent anxiety factors, derived from broad anxiety symptoms, with fasting. In this study anxiety disorder pathology was assessed at age 10, and disordered eating behaviour at age 14 (Schaumberg et al., 2019). Whether the reported associations are maintained over time, and particularly during the mid‐late adolescent period in which AN incidence is highest (Micali et al., 2013), remains unclear. Further, whether anxiety has a predictive influence on fasting behaviour over a shorter time period is unknown. Yet, understanding whether associations vary with developmental and predictive periods could elucidate mechanisms by which anxiety is related to fasting, to further understanding of AN aetiology.

The aim of the current study was to extend previous research by investigating the predictive influence of anxiety disorder pathology on fasting 2 years later, in mid‐late adolescence, in a large population cohort, the Avon Longitudinal Study of Parents and Children (ALSPAC). It was hypothesised that anxiety disorder presence would predict subsequent engagement in fasting.

## 2 METHOD

### 2.1 Data source

ALSPAC is a prospective population cohort study of families in the Bristol area of the United Kingdom (Boyd et al., 2013; Fraser et al., 2013). Mothers were eligible for the study if their expected dates of delivery were between April 1, 1991 and December 31, 1992, and 14,151 pregnant women were initially recruited. When the eldest child participants were aged seven an attempt to increase the sample was made. In the total sample, there were 15,247 pregnancies, 15,458 foetuses, and 14,701 children alive at 1‐year old. The ALSPAC study website contains details of all data collected from study participants. This is facilitated by use of the fully searchable variable catalogue and data dictionary (http://www.bristol.ac.uk/alspac/researchers/access/). Ethics approval for the study was obtained from the ALSPAC Ethics and Law Committee and the Local Research Ethics Committees.

### 2.2 Participants

We assessed whether the presence of an anxiety disorder at wave 13–14 and wave 15–16 predicted fasting at the subsequent wave (wave 15–16 and wave 17–18, respectively). Participants of the current study were individuals who had provided anxiety disorder and fasting data for at least one of the prospective analyses of interest. Participants also had to have provided data to indicate whether they engaged in fasting at baseline (wave 13–14) to be included. The timing of data collection, in terms of the developmental stage of participants, has been standardised across participants in ALSPAC as far as possible. At wave 13–14: median age of anxiety disorder assessment was 13 years, 10 months; fasting was assessed at 14 years. At wave 15–16: median age of anxiety disorder assessment was 15 years, 5 months; fasting was assessed at 16 years. At wave 17–18, adolescents were aged 18 when fasting was assessed. Participants remain eligible for follow‐up in ALSPAC unless they withdraw consent or are untraceable (Boyd et al., 2013). As a result, there are participants who have responded to assessment invitations at later, and not earlier, time‐points, and who are included in the second, but not first, longitudinal analysis of the study.

**Figure 1:**
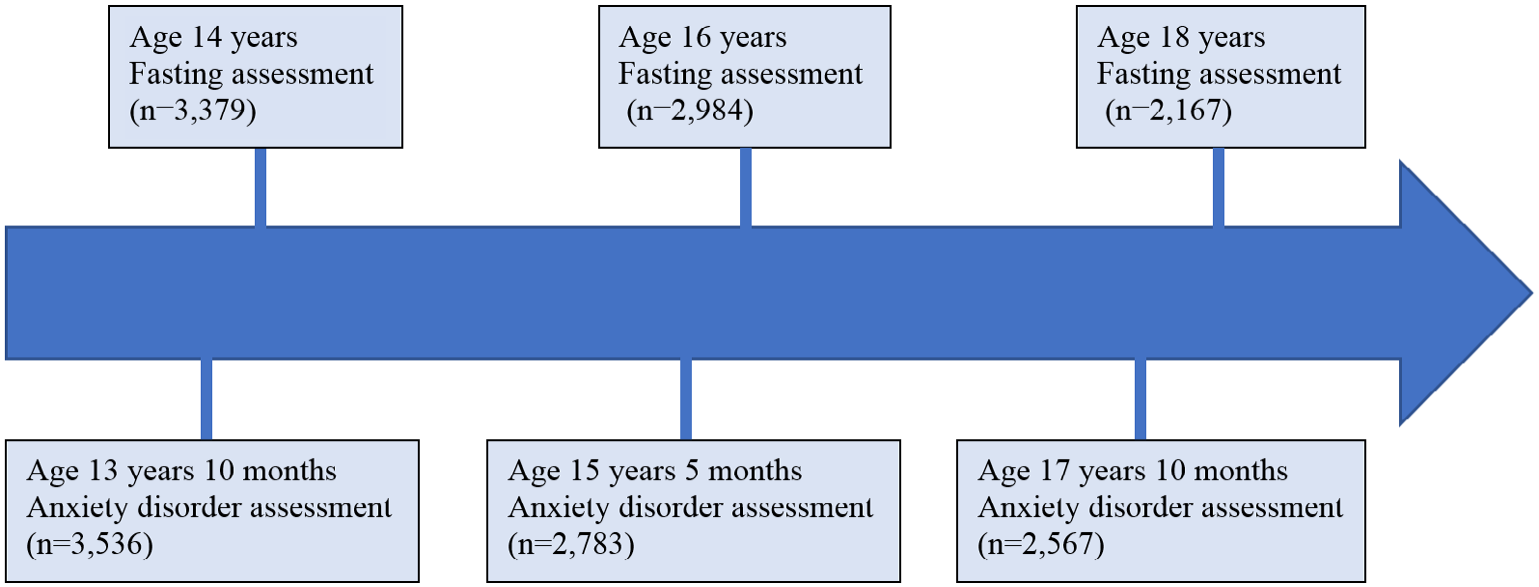
Diagram showing data collection process for the current investigation

Preliminary investigations found that both the exposure (anxiety disorder presence) and outcome (fasting) were extremely rare for males; in one analysis there were no males in the anxiety disorder and fasting category. Rare events and associated data sparseness affect the validity and precision of regression coefficient values (Greenland, Mansournia, & Altman, 2016; King & Zeng, 2001). The utility of a statistical test is also limited when there are no individuals in a category of interest. Given the data are not such to allow robust assessment of associations between anxiety disorders and fasting in males, even with methods designed to handle rare outcomes, we restricted our main analysis to females (*n* = 2,406). Figure 1 comprises a diagram of the data collection process, and of the number of participants at each stage.

### 2.3 Measures

#### 2.3.1 Outcome

Fasting for weight‐loss was assessed by response to the question “During the past year, how often did you fast (not eat for at least a day) to lose weight or avoid gaining weight?”. This question was based on one of the validated McKnight Risk Factor survey (Shisslak et al., 1999), and was posed to adolescents in mailed questionnaires. Participants could select from the following response options: less than once a month; monthly; weekly. The fasting outcome variable was a binary indicator of whether individuals had engaged in fasting behaviour on at least a monthly basis during the previous year. Monthly fasting has previously been used as a criterion to derive AN diagnoses (Micali et al., 2015a, 2015b), supporting use of the outcome when attempting to identify predictors of AN behaviour. Furthermore, monthly fasting reported at age 16 predicts subsequent AN diagnosis (at age 18) in females of the ALSPAC sample (Supporting Information, Table S1).

#### 2.3.2 Anxiety disorders

Anxiety disorder presence was assessed by the Development and Wellbeing Assessment (DAWBA; [Goodman, Ford, Richards, Gatward, & Meltzer, 2000]). The DAWBA comprises a package designed to generate psychiatric diagnoses based on criteria of the 10th revision of the International Statistical Classification of Diseases and Related Health Problems (ICD‐10; (World Health Organisation [WHO], 1992)) and the 4th edition of the Diagnostic and Statistical Manual of Mental Disorders (DSM‐IV (APA, 1994)). In the current study computer algorithms determined the likelihood of individuals experiencing an anxiety disorder using response data collected from semi‐structured interviews. There are six possible categories, ranging from level 0 (<0.1% chance of having an anxiety disorder), to level 5 (>70% chance of having an anxiety disorder). Individuals in the top two bands were at least 50% likely to have a given anxiety disorder and were assigned a diagnosis. This approach has been shown to produce anxiety disorder diagnoses that broadly align with clinician ratings (Goodman, Heiervang, Collishaw, & Goodman, 2011). At wave 13–14 adolescent symptoms were parent‐reported and the presence of generalised anxiety disorder, social phobia, specific phobia and separation anxiety disorder was assessed. At wave 15–16 adolescent symptoms were self‐reported, and the presence of generalised anxiety disorder, social phobia, specific phobia, panic disorder, and agoraphobia assessed.

Because of the rarity of anxiety disorders in the sample, we collapsed across diagnoses to create binary variables that indicated whether any of the assessed anxiety disorders were present, at each wave. These “any anxiety disorder” exposure variables were used in analyses of the current study. When classifying individuals according to anxiety disorder diagnosis, obsessive–compulsive disorder and PTSD were not considered since they are not included as anxiety disorders in current psychiatric diagnostic manuals.

#### 2.3.3 Co‐variates

Potential confounders of the anxiety disorder and fasting association were identified based on theory asserting the importance of particular variables in AN development, and previous findings regarding predictors of anxiety and restrictive eating. Specifically, these variables were: fasting at baseline (wave 13–14); binge‐eating at the earlier wave; purging at the earlier wave; and weight status (i.e., underweight, normal weight, overweight/obese) at the earlier wave. Prior research supports the proposal that unhealthy restrictive eating practices remain fairly stable throughout adolescence and young adulthood (Neumark‐Sztainer, Wall, Larson, Eisenberg, & Loth, 2011). Binge eating and purging are associated with both restrictive eating and anxiety symptoms (Lynch, Everingham, Dubitzky, Hartman, & Kasser, 2000; Micali, Horton, et al., 2017; Viborg, Wångby‐Lundh, Lundh, Wallin, & Johnsson, 2018). Cross‐sectional evidence supports greater restrictive eating amongst overweight/obese adolescents (Gonsalves, Hawk, & Goodenow, 2014; López‐Guimerà et al., 2013), while longitudinal studies have reported associations between childhood body mass index (BMI) and AN risk in both directions (Berkowitz et al., 2016; Yilmaz, Gottfredson, Zerwas, Bulik, & Micali, 2019).

Baseline fasting was assessed using the same question as that used to derive the outcome variable and indicated whether in the past year the individual had engaged in *any* fasting (i.e., less than monthly, or more frequently). Binge eating and purging were assessed in the same questionnaires that enquired about fasting behaviour at each of the waves, using questions adapted from those of the Youth Risk Behaviour Surveillance System Questionnaire (Kann et al., 1996). Binge eating was recorded if the adolescent reported episodes of eating large amounts of food while feeling out of control in the past year, purging was recorded if adolescents reported making themselves sick or using laxatives to lose weight/avoid gaining weight in the past year. The questions about binge eating and purging have been validated in an adolescent sample (Field, Taylor, Celio, & Colditz, 2004). Weight status was determined using age, gender and BMI information collected from adolescents, along with UK reference data (Cole, Freeman, & Preece, 1998) and cut‐offs defined by the WHO (WHO Expert Committee, 1995) and the International Obesity Taskforce (Cole, Bellizzi, Flegal, & Dietz, 2000). BMI was calculated using objective weight and height measurements taken during clinic assessments at each wave. At wave 13–14 self‐reported weight and height information was used when objective information was missing.

Univariable regression analyses assessed whether the potential confounders actually met criteria for confounding. Variables were considered confounders if they were associated with exposure (concurrent anxiety) and outcome (fasting 2 years later) to a threshold of *p* < .10 at either of the longitudinal waves. Baseline fasting, binge‐eating and purging met criteria for confounding (see Table S2) and were subsequently included as covariates in the main analysis.

Predictors of missing data were also included as covariates in the main analyses so as to satisfy missing data assumptions of statistical models (Lin & Rodriguez, 2015). Predictors of missingness in ALSPAC are the demographic variables social economic status (SES), mother age at delivery, and mother parity. SES was derived from the lowest social class of both parents (manual or non‐manual background). Mother parity was a binary indicator of whether the study child was the mothers’ first pregnancy to be carried to birth. Information in respect of these demographic variables was determined from questionnaire data.

### 2.4 Statistical analysis

All statistical analyses were conducted in Stata 14.1 (StataCorp, 2015a). Generalised estimating equation (GEE) models (Liang & Zeger, 1986) with an unstructured working correlation estimated the longitudinal association between anxiety disorder presence and odds of fasting at the subsequent wave, across both longitudinal waves (i.e., associations between anxiety disorder presence at wave 13–14 and fasting at wave 15–16, and between anxiety disorder presence at wave 15–16 and fasting at wave 17–18). The anxiety disorder exposure, and disordered eating covariates (binge eating and purging), were treated as time‐varying predictors, while all other covariates were time invariant. Models were adjusted for wave of assessment, and robust standard errors were calculated.

GEE models are an extension of the generalised linear model applied to repeated measures data. They take into account dependencies in the data to ensure accurate standard errors are generated, promoting correct inferences from analysis outcomes (Hanley, Negassa, Edwardes, & Forrester, 2003). Quasi‐likelihood, as opposed to maximum likelihood, estimation is employed; meaning specification of the outcome variable probability distribution is not required (Ghisletta & Spini, 2004; Zeger, Liang, & Albert, 1988). A function associated with the log‐likelihood, but not the log‐likelihood, is maximised to obtain parameter estimates. GEE models obtain population level effects, making the method suitable for the current study that seeks to determine the average effect of an anxiety disorder on fasting behaviour, rather than individual level effects (Hu, Goldberg, Hedeker, Flay, & Pentz, 1998).

To determine whether associations differed across the course of adolescence, we stratified the longitudinal data by wave of analysis and conducted binary logistic regression analyses. Models included the same predictors as the GEE analyses, and estimated coefficients using the maximum likelihood estimator.

We also investigated whether anxiety disorders predicted engagement in future fasting in the subpopulation of individuals who reported no fasting at baseline. The same method as that of the full sample analysis was used. Subsample analyses should be regarded as exploratory given the reduced sample size, and in particular the reduced number of cases.

Cross‐sectional analyses of associations between anxiety and fasting across three waves of data (i.e., wave 13–14, wave 15–16 and wave 17–18) were also completed. GEE and logistic regression models estimated associations across and within the waves respectively, as for the longitudinal analyses. Results of cross‐sectional analyses are available in Table S4.

#### 2.4.1 Sensitivity analyses

Sensitivity analyses assessed whether the association between anxiety disorders and fasting was specific, as opposed to reflecting an association between anxiety disorders and disordered eating more generally. We replicated original analyses in a sample that excluded individuals who reported fasting with concurrent binge eating or purging at the time of outcome assessment, and individuals who reported fasting and were missing binge eating or purging data. Outcomes from these analyses did not qualitatively differ from those of analyses that included the full sample. Similarly, results remained consistent when excluding from analyses participants who had responded to some but not all anxiety disorder diagnostic questions, which prevented diagnostic decisions over certain disorders. Standard errors were increased for restricted sample coefficient estimates, and coefficient estimates may themselves be inflated due to the increased rarity of the outcome event (Greenland et al., 2016; King & Zeng, 2001). As such, we report outcomes of analyses completed in the full sample.

### 2.5 Attrition

The availability of data varied with wave of analysis; at wave 13–14 the sample size was 2,204, and this reduced to 1,382 at wave 15–16. Missing covariate data was imputed using the multiple imputation by chained equation (MICE) approach, implemented with the multiple impute chained command in Stata (StataCorp, 2015b). MICE assumes data points are missing at random, and is suitable for data that is not multivariate normal (Aloisio, Swanson, Micali, Field, & Horton, 2014; Azur, Stuart, Frangakis, & Leaf, 2011). We created 70 imputed datasets. All variables of the analysis were included in the imputation models. Weight status variables from each of the waves, and anxiety disorder, binge eating and purging indicators at wave 17–18, were also included in imputation models given associations of these variables with analysis covariates.

## 3 RESULTS

### 3.1 Sample characteristics

Demographic information, and prevalence information for fasting, anxiety disorder and binge eating/purging variables is provided in Table 1. For a breakdown of anxiety disorder prevalence by anxiety disorder (see Table S3).

**Table 1:** Frequencies for demographic variables and anxiety disorder presence

Demographic variables	Frequencies
	*n* (%)
Parent lowest combined social class at enrolment^a^	
Manual	300 (12.47)
Non‐manual	1,696 (70.79)
Missing	410 (17.04)
Ethnicity	
White	2,108 (87.61)
Other ethnic group	37 (1.54)
Missing	261 (10.85)
Mother parity^b^	
Primipari	1,068 (44.39)
Multipari	1,180 (49.04)
Missing	158 (6.57)
Weight status	
Wave 13–14	
Underweight	212 (8.81)
Normal weight	1,516 (63.01)
Overweight/obese	382 (15.88)
Missing	296(12.30)
Wave 15–16	
Underweight	150 (6.11)
Normal weight	1,086 (45.14)
Overweight/obese	225 (9.35)
Missing	945 (39.28)
Wave 17–18	
Underweight	147 (6.11)
Normal weight	1,242 (51.62)
Overweight/obese	308 (16.96)
Missing	609 (25.31)
Fasting prevalence	
Wave 13–14	
Any fasting in past year	
Yes	217 (9.02)
No	2,189 (90.98)
Wave 15–16	
Monthly fasting in past year	
Yes	202 (8.40)
No	2,090 (86.87)
Missing	114 (4.74)
Wave 17–18	
Monthly fasting in past year	
Yes	124 (5.15)
No	1,554 (64.59)
Missing	728 (30.26)
Anxiety disorder prevalence^c^	
Wave 13–14	
Any anxiety disorder	
Yes	29 (1.21)
No	2,272 (94.43)
Missing	105 (4.36)
Wave 15–16	
Any anxiety disorder	
Yes	47 (1.95)
No	1,838 (76.39)
Missing	521 (21.65)
Covariate prevalence	
Wave 13–14	
Binge eating in past year	
Yes	163 (6.77)
No	1,976 (82.13)
Missing	267 (11.10)
Purging in past year	
Yes	53 (2.20)
No	2,341 (97.30)
Missing	12 (0.50)
Wave 15–16	
Binge eating in past year	
Yes	352 (14.63)
No	1,941 (80.67)
Missing	113 (4.70)
Purging in past year	
Yes	232 (9.64)
No	2,068 (85.95)
Missing	106 (4.41)

a Parent occupation is a proxy indicator for socio‐economic status, with manual and non‐manual occupations coded according to 1991 Office of Population, Censuses and Surveys classification.

b Parity describes whether the study child was the first carried to birth by the mother. Primipari indicates child was the first pregnancy carried to birth by the mother; multi‐pari indicates the mother had previous viable pregnancies.

c As indicated by Development and Wellbeing Assessment computer‐generated diagnostic bandings.

### 3.2 Associations between anxiety disorders and fasting

#### 3.2.1 Longitudinal analysis

GEE model estimates of effect and precision supported a longitudinal association between anxiety disorders and fasting, whereby anxiety disorder presence predicted an increased likelihood of engagement in fasting at the following wave (adjusted odds ratio [adjOR] = 2.07 [95% confidence interval (CI) 1.03, 4.17], *p* = .04). The statistical evidence for the association between anxiety disorder presence at wave 13–14 and fasting at wave 15–16 estimated by the logistic regression model was not strong (adjOR = 0.23 [95% CIs: 0.03, 1.83], *p* = .165). However, logistic regression model estimates indicated that individuals with an anxiety disorder at wave 15–16 were at greater risk of fasting at wave 17–18, and this was supported by the statistical evidence (adjOR = 6.38 [95% CIs: 2.81, 14.49], *p* < .001).

Findings of the exploratory GEE analysis supported anxiety disorder presence predicting increased likelihood of fasting at the following wave: adjOR = 2.61 [95% CIs:1.23, 5.54], *p* = .012. Outcomes of logistic regression models stratified by wave did not provide strong evidence for a predictive effect of anxiety disorders at wave 13–14 (adjOR = 0.58 [95% CI: 0.07, 4.47], *p* = .599). However, there was strong evidence to support anxiety disorder presence at wave 15–16 predicting engagement in fasting at wave 17–18 (adjOR = 6.41 [95% CI: 2.56, 16.05], *p* < .001).

Table 2 details full outcomes of longitudinal analyses, including coefficients of model covariates.

**Table 2:** Longitudinal associations of anxiety disorders and covariates with fasting

Outcome: Fasting for weight‐loss/to avoid weight‐gain at subsequent wave	Main analysis						Exploratory analysis that excludes individuals reporting any fasting at baseline					
			Logistic regression models stratified by wave						Logistic regression models stratified by wave			
	GEE model		Wave 13–14		Wave 15–16		GEE model		Wave 13–14		Wave 15–16	
Predictor variable	(*n* = 2,406)		(*n* = 2,204)		(*n* = 1,382)		(*n* = 2,406)		(*n* = 2,005)		(*n* = 1,276)	
	OR (95% CIs)	*p* value	OR (95% CIs)	*p* value	OR (95% CIs)	*p* value	OR (95% CIs)	*p* value	OR (95% CIs)	*p* value	OR (95% CIs)	*p* value
Anxiety	2.07 [1.03, 4.17]	.04	0.23 [0.03, 1.83]	.165	6.38 [2.81, 14.49]	<.001	2.61 [1.23, 5.54]	.012	0.58 [0.07, 4.47]	.599	6.41 [2.56, 16.05]	<.001
Fasting at 14	2.95 [2.09, 4.18]	<.001	3.41 [2.27, 5.1]	<.001	2.32 [1.28, 4.23]	.006	NA	NA	NA	NA	NA	NA
Binge eating	1.56 [1.03, 2.35]	.034	1.41 [0.83, 2.41]	.204	1.93 [1.15, 3.26]	.013	2.07 [1.34, 3.22]	.001	2.06 [1.15, 3.69]	.015	2.24 [1.28, 3.92]	.005
Purging	3.7 [2.45, 5.6]	<.001	2.9 [1.47, 5.74]	.002	5.41 [3.23, 9.05]	<.001	3.44 [2, 5.92]	<.001	2.46 [0.67, 9.04]	.175	4.89 [2.76, 8.67]	<.001
Socioeconomic status	1.09 [0.72, 1.64]	.687	0.92 [0.59, 1.44]	.714	1.59 [0.75, 3.4]	.227	1.00 [0.64, 1.57]	.995	0.84 [0.51, 1.39]	.505	1.45 [0.62, 3.39]	.39
Mother parity	1.36 [1.03, 1.8]	.031	1.38 [0.99, 1.92]	.054	1.26 [0.79, 2.03]	.334	1.31 [0.96, 1.78]	.083	1.31 [0.91, 1.88]	.15	1.28 [0.76, 2.13]	.352
Mother age at delivery	0.94 [0.91, 0.97]	<.001	0.94 [0.91, 0.98]	.002	0.93 [0.88, 0.98]	.009	0.94 [0.91, 0.98]	.004	0.95 [0.91, 0.99]	.013	0.94 [0.88, 1]	.035
Wave	0.67 [0.52, 0.86]	.002	NA	NA	NA	NA	0.67 [0.5, 0.89]	.005	NA	NA	NA	NA

## 4 DISCUSSION

The current study aimed to determine whether there was a longitudinal association between anxiety disorders and fasting for weight loss/to avoid weight‐gain in an adolescent sample. Findings partially supported our hypotheses. For females, across two longitudinal analyses, meeting diagnostic criteria for an anxiety disorder predicted an increased likelihood of fasting 2 years later, at the following wave. Exploratory analyses confirmed this association was present in a subset of individuals who did not engage in fasting at baseline. However, the prospective association observed was time‐sensitive: post‐hoc analyses (stratified by wave) confirmed that anxiety disorder presence predicted future fasting at wave 15–16, but not at wave 13–14. Outcomes of cross‐sectional analyses (Table S4) were similar to those of longitudinal analyses: anxiety disorder presence predicted an increased risk of concurrent fasting across three waves of data (waves 13–14, 15–16, 17–18), but the relationship was stronger at the latter two waves.

A recent study in the same population cohort identified associations of physical anxiety disorder symptoms assessed at age 10 with fasting behaviour at the 13–14 wave (Schaumberg et al., 2019). We build upon this finding to identify more proximal predictive effects of anxiety disorders on fasting, and effects beyond early adolescence, that is, the mid‐late adolescent period. The collection of findings suggests that the predictive influence of anxiety on fasting varies over time: anxious pathology in childhood and mid‐adolescence predicts increased risk of later fasting, while anxious pathology in early adolescence does not.

However, it is possible issues with the measurement of anxiety disorders at wave 13–14 prevented the detection of meaningful associations at this wave. Symptoms of anxiety disorders were parent‐reported at wave 13–14; non‐physical symptoms that could not be articulated by adolescents, or that did not have observable outcomes, may have gone unreported. In support of this, parental report of covert internalising symptoms, for example worry, is particularly discordant with child‐reported symptoms (Comer & Kendall, 2004). Parental assessment of child psychological symptoms tends not to correspond highly with child report generally however (De Los Reyes & Kazdin, 2005), with discrepancies increasing from childhood to adolescence (Achenbach, McConaughy, & Howell, 1987). Rather than simply being erroneous, it is possible parent‐reported symptoms reflect a different aspect of anxiety disorders as compared to self‐report, and one that is differentially associated with fasting. Indeed, previous studies support the value of multiple informants when assessing psychiatric pathology (Achenbach, Krukowski, Dumenci, & Ivanova, 2005), and have even indicated that parents may be the better source of information when making psychiatric assessments in some cases (Goodman et al., 2011; Kuhn et al., 2017). Future studies might consider using diagnostic information based on parent and child/adolescent reports when seeking to understand how anxiety disorder pathology may be associated with disordered eating outcomes.

Sensitivity analyses demonstrated that anxiety disorder presence predicted fasting in the absence of binge eating and purging. Dietary restriction intended to influence weight/shape, and not binge eating and purging, is the defining feature of AN, and required for diagnosis ‐ while the opposite is true for other eating disorders (APA, 2013). It could be that there is a stronger association between anxiety disorders and the eating pathology characteristic of AN, as compared to associations between anxiety and eating behaviour that is more typical of other eating disorders. This could explain why a previous cohort study (Zerwas et al., 2014), reported no predictive effect of anxiety disorders on disordered eating more generally (i.e., binge eating and purging in addition to restrictive eating).

Consistent with restrictive eating being characterised as a prodromal syndrome of AN (Jacobi et al., 2004), fasting predicted subsequent AN in the study sample. That anxiety disorders predict engagement in behaviour that is both indicative of increased AN risk and characteristic of AN (Aardoom et al., 2012; Linardon et al., 2018) aligns with outcomes of longitudinal studies in clinical populations. These studies consistently report anxiety disorders to precede, or to predict increased risk for, AN diagnosis (Bulik, Sullivan, Fear, & Joyce, 1997; Kaye et al., 2004; Meier et al., 2015). The associations that have been observed across various studies potentially lends support to the idea that individuals with non‐weight gain‐associated anxiety come to rely on dietary restriction as a means of managing this anxiety. Once the initial benefits of dietary restriction, in terms of anxiety regulation, are experienced, individuals may be driven to repeat the behaviour to the point of dependence (Godier & Park, 2014; Kaye et al., 2003; Kaye et al., 2009; Nunn et al., 2012). In this case, anxiety disorder pathology may be said to causally influence the development of eating patterns symptomatic and predictive of AN. However it is also possible that anxiety disorders signal the presence of an underlying predisposition to develop anxieties around weight‐gain and eating, and it is these that encourage engagement in severe forms of dietary restriction (Steinglass et al., 2011; Strober, 2004). In this case, non‐weight gain‐associated anxiety does not causally affect the development of restrictive eating behaviour, it simply highlights increased risk of engagement in such behaviour.

Parsing the two explanations apart to understand the relevance of anxiety disorders to disordered restrictive eating and AN is challenging. It has been found previously that adolescents with AN are more likely to later develop anxiety disorders compared to adolescents without AN (Micali et al., 2015b). The bidirectional associations that appear to exist between anxiety disorders and AN could reflect shared risk mechanisms. This perspective is supported by relatives of individuals with AN being more likely to have an anxiety disorder diagnosis compared to relatives of individuals without AN (Strober, Freeman, Lampert, & Diamond, 2007). Further, genetic correlations between generalised anxiety disorder and AN have been reported (Dellava, Kendler, & Neale, 2011), suggesting the two disorders share genetic risk factors. Alternatively, bidirectional associations between anxiety disorders and AN indicate the operation of a vicious cycle. Anxiety unrelated to weight‐gain may be dealt with initially by restrictive eating, encouraging the restrictive eating to continue. Adaptions within various neurobiological systems in response to limited food intake could then result in a resurgence of anxiety, elevating this beyond initial levels, to promote further engagement in dietary restriction (Kaye, 2008; Kaye et al., 2003). Understanding how anxiety disorders are associated with extreme forms of dietary restriction has implications for the development of effective eating disorder prevention and treatment interventions and should therefore be a priority of future research.

This study has a number of strengths. The large sample size and population‐based nature increase the validity and reliability of findings. The prospective design minimised risks of recall bias and reverse causation, and use of validated interviews in anxiety disorder assessment reduces potential measurement error. The GEE approach and calculation of robust standard errors enabled the correlation between participants’ repeated responses to be taken into account during the statistical analysis, promoting unbiased inferences.

It is also recognised that our study has limitations. First, the anxiety disorders assessed, and the informants of anxiety symptoms (i.e., parents or adolescents), differed by wave. This introduces challenges to directly comparing associations across the different waves. Second, DAWBA diagnoses based on computer‐generated bandings have been found previously to result in underestimation of disorder prevalence relative to clinician‐assigned DAWBA diagnoses. However, although reduced sensitivity to anxiety disorder pathology theoretically could introduce bias, effect estimates for associations of various factors with DAWBA psychiatric diagnoses do not differ according to whether diagnoses are computer‐generated or clinician‐assigned (Goodman et al., 2011). Third, the measure of fasting was questionnaire‐based, which may have affected measurement validity. This limitation also applies to the assessment of binge eating and purging. All disordered eating questions have been validated in previous studies however (Kann et al., 1996; Shisslak et al., 1999). Finally, findings may not generalise to other populations given ALSPAC participants are not representative of the UK population in terms of ethnicity. Since SES is a predictor of attrition in ALSPAC, findings may not extend to individuals from less advantaged backgrounds.

While findings can inform of the risk factors for fasting, with restrictive eating a prodromal syndrome and core symptom of AN, the knowledge generated from this study cannot be directly applied to AN. Fasting is more prevalent amongst adolescents and young adults than AN (Neumark‐Sztainer, Story, Hannan, Perry, & Irving, 2002; Neumark‐Sztainer, Wall, Eisenberg, Story, & Hannan, 2006), and many individuals who engage in the behaviour will never meet criteria for AN. In addition, our study conclusions cannot be extrapolated to males given male participants were not included in the final analysis. The rarity of fasting in male adolescents likely reflects differences in the presentation of disordered eating in males as compared to females. Excessive or compulsive exercise is more frequently endorsed, relative to restrictive eating, by male adolescents, which is not the case for females (Mond et al., 2014). Compulsive exercise has been reported to be more severe in males with AN as compared to females with the disorder (Murray, Griffiths, Rieger, & Touyz, 2014), suggesting exercise may be a core feature of AN pathology in males. It might be valuable for future research in community samples to consider excessive exercise outcomes when attempting to understand determinants of behaviour typical of AN in male populations.

Despite the discussed limitations, our study demonstrates that in females there is a predictive effect of anxiety disorders present in mid‐adolescence on subsequent fasting behaviour that is itself a risk factor for AN. Findings highlight anxiety disorder pathology as a potential target for eating disorder prevention efforts, although further research is required to determine the mechanisms underlying the observed association. Advances in the understanding of genetic risk factors and neurocognitive antecedents/outcomes of anxiety disorders and AN may elucidate the nature of the relationship between anxiety and restrictive eating, with studies of experimental design testing hypotheses surrounding causation.

## Supporting information


**Table S1**. Prospective prediction of anorexia nervosa diagnosis at wave 17–18 by monthly fasting at wave 15–16.
**Table S2**. Associations of potential confounders with anxiety disorder exposure and fasting outcome.
**Table S3**. Frequencies for anxiety disorder diagnoses in the study population.

**Table S4** Cross‐sectional associations of anxiety disorders with fasting.
