# Supplementary material for: Anxiety disorders predict fasting to control weight: A longitudinal large cohort study of adolescents
Source: Eur Eat Disord Rev. 2019 Dec 17;28(3):269–81. doi: 10.1002/erv.2714 (PMC7192761; doi:10.1002/erv.2714)
Supplement: Supplementary file 1 — Table S1. Prospective prediction of anorexia nervosa diagnosis at wave 17–18 by monthly fasting at wave 15–16. Table S2. Associations of potential confounders with anxiety disorder exposure and fasting outcome. Table S3. Frequencies for anxiety disorder diagnoses in the study population. [file ERV-28-269-s001.docx]

**Supplementary Material 1**

**Table 1 Prospective Prediction of Anorexia Nervosa Diagnosis at Wave 17-18 by Monthly Fasting at Wave 15-16**

| Outcome: Anorexia Nervosa Diagnosis at Wave 17-18  (*n* = 1552) | Logistic Regression Model Estimate | |
| --- | --- | --- |
|  | OR [95% CIs] | P value |
| Monthly Fasting at Wave 15-16 | 3.35[1.09, 10.26]* | 0.034 |

Individuals meeting criteria for anorexia nervosa at wave 13-14 or wave 15-16 were excluded from the analysis.

**Table 2 Associations of Potential Confounders with Anxiety Disorder Exposure and Fasting outcome**

|  | Outcome | | | | | | |
| --- | --- | --- | --- | --- | --- | --- | --- |
|  | **Anxiety disorder at same wave** | | | **Fasting at subsequent wave** | | | |
|  | OR  [95% CI] | P value | *N* | | OR  [95% CI] | p | *N* |
| Predictor |  |  |  | |  |  |  |
| *Wave 13-14* |  |  |  | |  |  |  |
| Binge eating | 3.01  [1.00,9.05] | 0.05 | 2047 | | 1.75  [1.06,2.88] | 0.028 | 2040 |
| Purging | 3.42  [0.79,14.77] | 0.1 | 2290 | | 6.07  [3.30,11.18] | <0.001 | 2281 |
| Weight status | 0.91  [0.47,1.76] | 0.777 | 2110 | | 1.56  [1.24,1.97] | <0.001 | 2019 |
| *Wave 15-16* |  |  |  | |  |  |  |
| Binge eating | 3.69  [1.85,7.34] | <0.001 | 1779 | | 6.85  [4.46,10.52] | <0.001 | 1572 |
| Purging | 1.41  [0.64,3.07] | 0.393 | 1772 | | 3.02  [1.99,4.57] | <0.001 | 1566 |
| Weight status | 1.61  [0.87,2.98] | 0.129 | 1179 | | 1.4  [0.92,2.13] | 0.119 | 1035 |

For predictors at wave 13-14, the subsequent wave is wave 15-16; for predictors at wave 15-16, the subsequent wave is wave 17-18.

**Table 3 Frequencies for Anxiety Disorder Diagnoses in the Study Population**

|  | Wave 13-14 | | | Wave 15-16 | | |
| --- | --- | --- | --- | --- | --- | --- |
|  | *N* (%) | | | *N* (%) | | |
|  | Yes | No | Missing | Yes | No | Missing |
| Generalized Anxiety Disorder | 6  (0.25) | 2,261 (93.97) | 139 (5.78) | 19 (0.79) | 1,864 (77.47) | 523 (21.74) |
| Social Phobia | 13 (0.54) | 2,285 (94.97) | 280  (11.64) | 20 (0.83) | 1,865 (77.51) | 521 (21.65) |
| Specific Phobia | 11 (0.46) | 2,290 (95.18) | 105 (4.36) | 12 (0.50) | 1,871 (77.76) | 523 (21.74) |
| Separation Anxiety Disorder (DSM-IV) | 13 (0.54) | 2,133 (87.82) | 280 (11.64) |  |  |  |
| Separation Anxiety Disorder (ICD-10) | 11 (0.46) | 2,115  (87.91) | 280 (11.64) |  |  |  |
| Panic Disorder |  |  |  | <5  (0.17) | 1,877 (78.01) | 525 (21.82) |
| Agoraphobia |  |  |  | 6  (0.25) | 1875 (77.93) | 525 (21.82) |
| Any anxiety disorder | 42 (1.39) | 2980 (98.61) |  | 47 (1.95) | 1,838 (76.39) | 521 (21.65) |
