# Supplementary material for: Anxiety disorders predict fasting to control weight: A longitudinal large cohort study of adolescents
Source: Eur Eat Disord Rev. 2019 Dec 17;28(3):269–81. doi: 10.1002/erv.2714 (PMC7192761; doi:10.1002/erv.2714)
Supplement: Supplementary file 2 — Table S4 Cross‐sectional associations of anxiety disorders with fasting. [file ERV-28-269-s002.docx]

**Cross Sectional Analyses**

**Table 1 Cross-sectional Associations of Anxiety Disorders with Fasting**

| **Outcome: Concurrent fasting for weight-loss/to avoid weight-gain** | **GEE model** | | **Logistic regression models stratified by wave** | | | | | |
| --- | --- | --- | --- | --- | --- | --- | --- | --- |
| **Predictor variable** | (*n* = 2396) | | Wave 13-14^a^  (*n*=2301) | | Wave 15-16  (*n*=1,771) | | Wave 17-18^b^  (*n*=1,265) | |
|  | OR  [95% CIs] | P value | OR  [95% CIs] | P value | OR  [95% CIs] | P value | OR  [95% CIs] | P value |
| Anxiety^c^ | 2.95  [1.9, 4.58] | <0.001 | 1.82  [0.64, 5.12] | 0.259 | 5.07  [2.37, 10.89] | <0.001 | 3.01  [1.76, 5.16] | <0.001 |
| Binge eating^c^ | 2.37  [1.79, 3.14] | <0.001 | 3.53  [2.28, 5.47] | <0.001 | 2.14  [1.4, 3.28] | <0.001 | 1.86  [1.1, 3.14] | 0.02 |
| Purging^c^ | 8.88  [6.52, 12.1] | <0.001 | 19.61  [10.47, 36.75] | <0.001 | 9.08  [6.09, 13.53] | <0.001 | 8.92  [5.25, 15.16] | <0.001 |
| Socio-economic status | 0.71  [0.52, 0.99] | 0.04 | 0.65  [0.42, 1.01] | 0.056 | 0.67  [0.4, 1.14] | 0.14 | 1.29  [0.57, 2.9] | 0.542 |
| Mother parity | 1.34  [1.05, 1.71] | 0.018 | 1.25  [0.88, 1.78] | 0.219 | 1.51  [1.01, 2.25] | 0.044 | 1.13  [0.68, 1.9] | 0.633 |
| Mother age at delivery | 0.97  [0.94, 1] | 0.038 | 1  [0.96, 1.03] | 0.853 | 0.94  [0.9, 0.98] | 0.007 | 0.95  [0.9, 1.01] | 0.129 |
| Wave | 0.63  [0.54, 0.73] | <0.001 | NA | NA | NA | NA | NA | NA |

^a^ At wave 13-14 the association of anxiety and covariates with any fasting in the past year is assessed, unlike at the later waves, where the association of anxiety and covariates with monthly fasting during the past year is assessed.

^b^ At wave 17-18 anxiety disorder presence was assessed with the Clinical Interview Schedule-Revised ((CIS-R)((Lewis, Pelosi, Araya, & Dunn, 1992)) rather than the DAWBA (Goodman, Ford, Richards, Gatward, & Meltzer, 2000). The CIS-R is a semi-structured computerized assessment of psychopathology that yields symptom presence indicators and diagnoses based on ICD-10 criteria.

^c^ Treated as time-varying predictor in the Generalized Estimating Equation model, such that the effect estimate reflects the concurrent association between the variable and the fasting outcome across the three cross-sectional waves of data.

**References**

Goodman, R., Ford, T., Richards, H., Gatward, R., & Meltzer, H. (2000). The Development and Well-Being Assessment: description and initial validation of an integrated assessment of child and adolescent psychopathology. *J Child Psychol Psychiatry, 41*(5), 645-655.

Lewis, G., Pelosi, A. J., Araya, R., & Dunn, G. (1992). Measuring psychiatric disorder in the community: a standardized assessment for use by lay interviewers. *Psychol Med, 22*(2), 465-486.
